# Advances and Persistent Challenges in the Management of Necrotizing Soft Tissue Infections: Time for a Systems-Level Approach

**DOI:** 10.3390/bioengineering13040443

**Published:** 2026-04-10

**Authors:** Marcelo A. F. Ribeiro, Sharon Henry

**Affiliations:** R Adams Cowley Shock Trauma Center, University of Maryland School of Medicine, Baltimore, MD 21201, USA

Necrotizing soft tissue infections (NSTIs) remain one of the most unforgiving diseases encountered in modern surgical practice. Despite decades of advances in critical care, antimicrobial therapy, and surgical technique, outcomes have improved only marginally. Mortality remains unacceptably high, and survivors often face devastating functional consequences. This reality forces us to confront an uncomfortable truth: while our knowledge has evolved, our systems of care have not kept pace. The current incidence of necrotizing soft tissue infections (NSTIs) ranges from 0.2 to 6.9 per 100,000 person-years globally, with significant geographic variation. In the United States specifically, recent estimates indicate an incidence of 8.7 to 10.3 per 100,000 persons annually, which is higher than previously reported and likely reflects improved case identification [1,2].

At the University of Maryland R Adams Cowley Shock Trauma Center, where we manage a high volume of complex soft tissue infections, NSTIs consistently demand rapid judgment, decisive action, and coordinated multidisciplinary care. These cases leave little margin for hesitation. The disease progresses faster than our traditional diagnostic frameworks, and reliance on laboratory or imaging confirmation often comes at the cost of time—the single most critical determinant of survival.

Recent international efforts, including the Global Alliance for Infections in Surgery position statement, reinforce what experienced clinicians have long recognized: a clinical diagnosis of NSTI requires immediate action [3]. The emphasis on early source control, broad-spectrum antibiotics, and aggressive resuscitation is not new—but the persistent failure to deliver these interventions in a timely and coordinated manner remains a central problem. In practice, delays in recognition and surgical intervention continue to drive mortality.

One of the most striking—and concerning—observations is that NSTIs are still frequently misdiagnosed in their early stages. Even in experienced centers, the overlap with less severe infections can obscure the diagnosis. Scoring systems and laboratory markers have been proposed, but none have proven sufficiently reliable to guide decision-making independently. In our experience, which is supported by the literature, these tools should never delay operative exploration when clinical suspicion exists.

Another important contribution within this Special Issue is the review by Tran et al., which critically evaluates the current landscape of predictive models for NSTIs and highlights a fundamental limitation in our field—the absence of a reliable, universally applicable diagnostic or prognostic scoring system [4]. Despite the widespread use of tools such as LRINEC, the authors demonstrate that these models lack sufficient sensitivity and should not be used in isolation, reinforcing the primacy of clinical judgment. More contemporary approaches, including the NECROSIS score and machine learning-based models such as POTTER, show conceptual promise but remain limited by a lack of external validation or poor performance in NSTI-specific populations. Notably, the exploration of simple inflammatory markers such as NLR and PLR underscores the ongoing effort to identify accessible, early indicators of disease severity, although their clinical utility remains inconsistent. Collectively, this work emphasizes that NSTIs are too complex to be captured by single-parameter or static models and supports the need for integrative, dynamic, and potentially AI-driven approaches that incorporate clinical, laboratory, and patient-specific variables—an evolution that aligns with the broader shift toward precision and systems-based care in acute surgical infections.

Our group’s contribution to this Special Issue further highlights the limitations of traditional diagnostic approaches. The low yield of blood cultures in transferred patients, particularly after prior antibiotic exposure, raises important questions about the routine use of such tests in this population [5]. More importantly, it reinforces the need to shift our focus away from confirmatory diagnostics and toward early, decisive intervention.

Antimicrobial therapy, while essential, is often overemphasized relative to its true impact. Antibiotics do not penetrate necrotic tissue effectively, and their role is supportive rather than definitive. Early administration remains critical, ideally within the first hour of suspicion, but without timely surgical debridement, even the most appropriate antimicrobial regimen will fail. The challenge moving forward lies not only in selecting the right antibiotics but also in optimizing their delivery in critically ill patients, where altered pharmacokinetics can significantly impact efficacy [6].

Surgery remains the cornerstone of treatment—this has not changed and never will. What has evolved is our understanding of how aggressive and how early that intervention must be. The concept of a single “definitive” operation has given way to a strategy of staged, iterative debridement. In our practice, repeated exploration—often within 24 h—is not the exception but the rule. This approach aligns with contemporary recommendations emphasizing serial source control as a dynamic process rather than a one-time event [1].

However, focusing solely on debridement overlooks a critical aspect of NSTI care: reconstruction. The magnitude of tissue loss following adequate source control is often substantial, and the pathway to functional recovery is complex. This Special Issue underscores the importance of integrating reconstructive strategies early in the treatment course, from negative pressure wound therapy to advanced flap techniques [7]. Reconstruction is not an afterthought—it is an essential component of care that directly impacts long-term outcomes.

At the same time, complications related to reconstruction—particularly vascular compromise—remain poorly understood and insufficiently studied [8]. As survival improves, these downstream challenges will become increasingly relevant, and thus they deserve greater attention within the surgical community.

Emerging technologies, including nanomedicine and advanced biomaterials, offer exciting possibilities but remain largely experimental. While early data suggest potential benefits in infection control and wound healing, their clinical role has yet to be defined [9]. As with many surgical innovations, the challenge will be translating promising concepts into reproducible, evidence-based practices.

Perhaps the most important—and least discussed—aspect of NSTI management is the organization of care. These patients require a level of coordination that extends beyond individual expertise. Multidisciplinary teams, standardized protocols, and concentration of care in experienced centers are not luxuries; they are necessities. The concept of dedicated NSTI teams or referral networks, as suggested in recent international recommendations, represents a logical and necessary evolution in care delivery [1,2,3,9,10].

From our perspective, improving NSTI outcomes will depend less on discovering new therapies and more on optimizing the delivery of the therapies we already have. Early recognition, immediate surgical intervention, appropriate antimicrobial therapy, and coordinated multidisciplinary care are well-established principles. The challenge lies in executing them consistently and without delay.

NSTIs do not tolerate hesitation. Neither should we.
